# Anaesthetic management of patients undergoing deep brain simulation: A retrospective review of 8 cases from a tertiary care center of Pakistan

**DOI:** 10.12669/pjms.36.7.2870

**Published:** 2020

**Authors:** Usama Ahmed, Faraz Shafiq, Dileep Kumar, Khalid Ahsan, Waleed Bin Ghaffar, Ehsan Bari

**Affiliations:** 1 Usama Ahmed, Resident, Department of Anesthesiology, The Aga Khan University, Karachi, Pakistan; 2 Faraz Shafiq, Assistant Professor, Department of Anesthesiology, The Aga Khan University, Karachi, Pakistan; 3 Dileep Kumar, Assistant Professor, Department of Anesthesiology, The Aga Khan University, Karachi, Pakistan; 4 Khalid Ahsan, Assistant Professor, Department of Anesthesiology, The Aga Khan University, Karachi, Pakistan; 5 Waleed Bin Ghaffar, Resident, Department of Anesthesiology, The Aga Khan University, Karachi, Pakistan; 6 Ehsan Bari, Associate Professor, Department of Surgery, The Aga Khan University, Karachi, Pakistan

**Keywords:** Anaesthetic, Parkinsonism, Deep Brain Stimulation, Pakistan

## Abstract

**Objectives:**

To review anaesthesia related outcome, perioperative complications and overall length of stay (LOS) in hospital for patients who had deep brain stimulation (DBS).

**Methods:**

The study was retrospective review of patients medical records diagnosed with Parkinson disease (PD) and underwent DBS at The Aga Khan University Hospital, Karachi from 2017-2019. Data was reviewed from file notes and patient chart and recorded on predesigned Performa. Frequency and percentages were used to present the data.

**Results:**

All patients were anaesthetized using Sleep-Awake-Sleep technique (SAS). Dexmedetomidine was mainly used for conscious sedation. Bispectral index monitor (BIS) was used to monitor the depth of sedation, and kept between 70-85 during sedative phase. All patients had successful intraoperative neurological monitoring, stimulation, and placement of electrodes. Total duration of anesthesia varied significantly in between the patients. Maximum duration was 600 minutes. None of our patient had any intraoperative event related to anaesthetic management. Overall five patients had some adverse events during ward stay. Mean LOS in hospital was four days.

**Conclusion:**

Anaesthetic management of DBS is well-tolerated. It requires dedicated team. The SAS technique is excellent for intraoperative neurophysiological monitoring. Careful selection of sedative agents and monitoring depth of anaesthesia using BIS would be beneficial in terms of improving related outcomes.

## INTRODUCTION

Parkinson’s disease (PD) is one of the commonest degenerative neurological diseases.^1^ Incidence of PD has been estimated to be 4.5-21 cases per 100,000 population per year.^2^ Pathophysiology of the disease is loss of dopaminergic neurons in substantia nigra, resulting in imbalance between dopamine and cholinergic neurons. Diagnosis is usually clinical, based on symptoms of bradykinesia, rigidity and tremors.^3^ Medical management aims to control signs and symptoms related to disease.^4^ However, in severe cases, poor control or extreme side effects may occur due to medical treatment. Deep brain stimulation (DBS) is indicated for such patients, as an effective measure to improve quality of life.^5^

DBS is actually an invasive procedure, involving placement of electrode and stimulation of thalamic nuclei surgically. Target nuclei (Subthalamic nuclei, Globus pallidus, Pars internal, Ventralis and intermedius) lie deep. This requires coordinated system of MRI guided stereo-tactic frame placement and location of target using computer software. Insertion of electrodes requires bilateral burr-hole drilling followed by electrodes insertion towards the planned trajectory. The process is guided both by micro-electrical recordings (MER) and macro-stimulation (MAS) to see patient’s response. This is followed by implantation of generator subcutaneously under the upper chest wall.^6^

Anaesthetic management of adult DBS requires neuro-anaesthesia team skilled in provision of sleep awake sleep technique (SAS). This needs preoperative psychological counseling and patients preparation like any awake neurosurgical technique.^7^ Anaesthesia related concerns of PD, associated medical therapy and principles of conscious sedation need to be considered. Goal is to keep patient comfortable, pain free, and provision of stable hemodynamics.^8^ This also includes facilitation of intraoperative MER and MAS, which is crucial for the successful placement of electrodes. In Pakistan, Neuro-anesthetic services related to DBS are yet in initial phases. The technique is fairly new to our part of world. Over last three years, we have done only eight cases. Besides, being a resource limited country, we are still struggling with availability of resources and skill required for this technique.

The primary objective of this retrospective study was to see anaesthesia related outcome of patients had DBS at our tertiary care hospital. This included review of anaesthesia technique and intraoperative complications. Secondary objective was to record complications that occurred in postoperative period, length of stay (LOS) at post anaesthesia care unit (PACU), special care unit (SCU) and overall LOS in hospital.

## METHODS

The study was retrospective review of patient’s medical record diagnosed with PD and underwent DBS at The Aga Khan University Hospital, Karachi. Study was conducted after getting approval from hospital’s ethical review committee (Approval ID: 2020-4745-10372). Data from of January 2017 to December 2019 was retrieved from health information and management system of our hospital.

Each patient’s base line demographic data including age, sex, American Society of Anesthesiologists (ASA) physical status and comorbid conditions were recorded using predesign Performa. Severity of PD and indication of surgical treatment was also noted. Information related to anaesthetic management; technique used, intraoperative monitoring, analgesic regime, medications used for conscious sedation, success in terms of MER and MAS was also recorded. Perioperative complications like airway related events, nausea, vomiting, and seizures were reviewed. The duration of stay in PACU, and SCU and overall LOS in the hospital was also monitored.

### Statistical Analysis:

The data was analyzed using frequencies, mean, and percentages using SPSS version 12. Results are presented using tables.

## RESULTS

Data of eight patients had DBS from 2017-19 was analyzed. Primary surgical indication was severe Parkinsonism associated with marked limitation in daily activities. According to Hoehn and Yahr scale,^9^ 75% patients were labeled to have stage IV, and two patients (25%) had stage III disease. All patients (100%) had preoperative anaesthesia assessment and psychological preparation by neuro-anaesthetist. Demographic characteristics of these patients including comorbid conditions are shown in Table-I.

**Table-I:** Demographic details of patients.

Patient	Age (Years.)	Sex (M/F)	ASA status	Comorbidities
1	52	M	3	-
2	55	M	3	-
3	57	M	3	-
4	40	M	3	-
5	53	M	3	-
6	60	M	3	-
7	40	F	3	Bipolar Disorder
8	56	M	3	Hypothyroidism

The anaesthetic technique was SAS technique in all (100%) patients. Routine ASA specific monitoring including noninvasive blood pressure, oxygen saturation, and electrocardiogram was done in all patients. Local anaesthetic infiltration was used for the placement of head frame in MRI suite. Conscious sedation was employed using either Propofol (1-3mg/Kg/hour) or Dexmedetomidine (0.1-1 ug/Kg/hour). Depth of sedation was monitored using Bispectral index monitoring (BIS) in all of these patients. BIS level was kept between 70-85. One patient did not require sedation because of excessive use of diazepam preoperatively. Supplemental analgesia was provided by intravenous fentanyl boluses of 10 micrograms (mcg). All patients had successful intraoperative MER, MAS and the placement of electrodes. For general anaesthesia (GA), airway was maintained with supra-glottic airway (SGA)device in two patients (25%), while endotracheal intubation was done in six patients (75%). Total duration of anesthesia varied significantly between 312 to 600. There was no intraoperative event of desaturation, seizure, nausea or vomiting. All patients were shifted to PACU, where their stay was uneventful and then signed out to SCU. Overall, five patients had some adverse events during the ward stay depicted Table-III. One patient required adjustment of the electrodes under local anesthesia due to improper signal delivery at the target site. Two patients had anxiety/depression and psychosis postoperatively. The mean duration of stay of these patients in SCU and ward was 2 days respectively; while overall LOS in hospital was 4 days.

**Table-II:** Anaesthetic Management.

Patient	Sedative Medication: Propofol/Dexmedetomidine infusion	Total Fentanyl Consumption (ug)	Airway management SGA/ETT	Duration (Minutes)
1	-	125	ETT	370
2	Dexmedetomidine	160	ETT	375
3	Dexmedetomidine	185	SGA	540
4	Dexmedetomidine	240	SGA	600
5	Dexmedetomidine	190	ETT	510
6	Propofol	200	ETT	295
7	Propofol	240	ETT	312
8	Dexmedetomidine	160	ETT	480

**Table-III:** Post procedural complications.

Patient	Postoperative Adverse Events n=5
1	Lower limb stiffness
2	None
3	Repositioning of electrodes
4	Temporary dyskinesia
	Drowsiness
	Left eye ptosis
5	None
6	None
7	Post DBS depression
8	Acute psychosis
	Agitation
	Dyskinesia

## DISCUSSION

This retrospective review describes anesthetic management of DBS. The chosen SAS technique worked well for all of our cases. These patients need assessment, optimization and psychological preparation preoperatively. The skilled anaesthetist would be the best person to deal with this. It is routine at our setup, to have Neuro anaesthetist based preoperative assessment for patients having awake craniotomy and DBS. A short power point presentation, which included detail description of anaesthesia technique was used for psychological preparation of these patients. Most challenging part is initial phase of surgery, where patient requires conscious sedation. This intermittent sedative state is required for bilateral insertion of stimulating electrodes and patient awakening during MER and MAS. For two of our cases (29%) we used Propofol for sedation. However, after having recent availability of Dexmedetomidine in Pakistan, most of our cases (71%) were managed with its infusion. Centrally acting alpha-2 receptor mediated sedation and analgesia without any respiratory depression make Dexmedetomidine, a preferable agent for conscious sedation in this scenario. Its collaborative sleep induction pattern, allows better recovery profile and responsiveness after discontinuation of infusion.^10^

Intraoperative phase is not risk free. Adverse events at this stage can be airway related, anxiety, vomiting and seizures. Institution of GA on emergency basis is considered as failure and may cause complications and adverse neurological outcome. Airway related events are mostly associated with the use of Propofol alone, or in combination with Remifentanil.^11^ None of our case had any adverse event intraoperatively. This may be due to tighter monitoring of sedation level. BIS guided sedation is proven to be beneficial not only in terms of titration of anesthetics but also for better recovery profiles.^12^

As part of preoperative preparation anti-Parkinson’s drugs need to be discontinued night before surgery It is important for intraoperative neurological monitoring. However, one should anticipate worsening of symptoms on operative day. One of our patient had severe dystonia, aggravated at the time of placing stereotactic frame. This required multiple boluses of diazepam (Total 10mg). However, it was associated with deep sedation throughout the duration of surgery. Careful management of symptoms is warranted when such emergencies occur. First line treatment is with benztropine 1-2 mg IV. Antihistamines with anticholinergic activity e.g., diphenhydramine, promethazine can be used alternatively.^13^

Pain management was done using multimodal analgesic regime. This included local infiltration at the site of burr hole and intraoperative fentanyl boluses. Cumulative fentanyl consumption is mentioned in Table-II. Fentanyl was mainly used at time of GA. Post procedural analgesia was prescribed using intermittent doses of IV Paracetamol and Tramadol, as per our Neuro-anaesthesia protocol.^14^

All of our patients had uneventful stay at PACU. Double antiemetic prophylaxis using Dexamethasone and Ondansetron worked very well throughout the perioperative period in our patients. Overall, we identified six adverse events in our patients postoperatively. None of these events were associated with prolong SCU or hospital stay. Post procedure, patients had remarkable improvement of symptoms and are in the follow-up. Information about the neurological outcome was not the objective of this retrospective review. The results of SAS technique are promising and showed convincing results in our retrospective review.

### Limitations of the Study

Considering the small sample size and retrospective nature of this review any recommendation at this point cannot be reinforced.

## CONCLUSION

Anaesthetic management of DBS is well-tolerated. This requires dedicated team with special interest in Neuro-anaesthesia. The SAS technique is excellent for intraoperative neurophysiological monitoring. Careful selection of sedative agents and monitoring depth of anaesthesia using BIS would be really beneficial in terms of overall anesthetic outcome related to the procedure.

### Author`s Contribution:

**UA:** Manuscript writing, Formatting. He is also the responsible and accountable for this study.

**FS:** Conceived, Design, Manuscripts writing,

**DK:** Data Collection.

**KA:** Data Collection.

**WBG:** Data Analysis.

**EB:** Manuscript writing.
